# Permanent farnesylation of lamin A mutants linked to progeria impairs its phosphorylation at serine 22 during interphase

**DOI:** 10.18632/aging.100903

**Published:** 2016-02-21

**Authors:** Olga Moiseeva, Stéphane Lopes-Paciencia, Geneviève Huot, Frédéric Lessard, Gerardo Ferbeyre

**Affiliations:** ^1^ Department of Biochemistry and Molecular Medicine, Université de Montréal, Montréal, Québec, H3C 3J7, Canada

**Keywords:** lamin A, cyclin dependent kinases, senescence, liquid droplets, Hutchinson-Gilford progeria syndrome

## Abstract

Mutants of lamin A cause diseases including the Hutchinson-Gilford progeria syndrome (HGPS) characterized by premature aging. Lamin A undergoes a series of processing reactions, including farnesylation and proteolytic cleavage of the farnesylated C-terminal domain. The role of cleavage is unknown but mutations that affect this reaction lead to progeria. Here we show that interphase serine 22 phosphorylation of endogenous mutant lamin A (progerin) is defective in cells from HGPS patients. This defect can be mimicked by expressing progerin in human cells and prevented by inhibition of farnesylation. Furthermore, serine 22 phosphorylation of non-farnesylated progerin was enhanced by a mutation that disrupts lamin A head to tail interactions. The phosphorylation of lamin A or non-farnesylated progerin was associated to the formation of spherical intranuclear lamin A droplets that accumulate protein kinases of the CDK family capable of phosphorylating lamin A at serine 22. CDK inhibitors compromised the turnover of progerin, accelerated senescence of HGPS cells and reversed the effects of FTI on progerin levels. We discuss a model of progeria where faulty serine 22 phosphorylation compromises phase separation of lamin A polymers, leading to accumulation of functionally impaired lamin A structures.

## INTRODUCTION

The nuclear lamina is a fibrous arrangement underneath the inner nuclear membrane that plays an important structural role determining the mechanical properties of the nucleus [1,2]. One of the main components of lamina is the intermediate filament protein lamin A [3]. Lamin A expression increases during cell differentiation [4] and in addition to its role at the nuclear membrane, it localizes to the nucleoplasm [5] to regulate DNA replication, transcription, and protein-protein interactions [6,7]. During cell division, the filamentous structure of the nuclear lamina is disassembled at prometaphase and reassembled after cytokinesis [8]. Assembly of nuclear lamina starts from longitudinal head-to-tail associations of lamin A soluble dimers. The resulting polymers associate laterally into fibers, and finally form paracrystals [9]. These polymers are resistant to harsh extraction conditions [10] and has been modelled as an elastic solid, resistant to deformation [11]. Both the N- and C-terminus of lamin A controls the solubility of the protein [12] and deletions of either the N-terminus head domain or the C terminus CaaX farnesylation domain impair localization to the nuclear lamina, leading to the formation of intranuclear lamin A aggregates [13]. Lamina assembly and disassembly is regulated during mitosis by Cdk1-dependent phosphorylation at both the N-terminus and the C-terminus [14]. Phosphorylation does not affect lamin dimerization but inhibits the longitudinal head to tail associations [15]. In addition to mitotic phosphorylation, lamin A is also phosphorylated in interphase at multiple sites including the sites phosphorylated during mitosis [16].

Lamin A is expressed as prelamin A and undergoes post-translational modifications, including farnesylation of C-terminal CaaX motif, endoproteolytic cleavage of the last three amino acids, methylation of C-terminal cysteine, and a second C-terminal endoproteolysis that removes the farnesyl group [6]. Farnesylation of prelamin A is a critical step for targeting the protein to the nuclear membrane [17] but the role of the last endoproteolysis performed by ZMPSTE24 is unknown. Different lamin A mutations lead to development of a wide range of diseases, termed laminopathies [3]. The most severe laminopathy is the Hutchinson-Gilford progeria syndrome (HGPS), which is characterized by premature aging and includes slow growth, loss of hair, lipodystrophy, and arteriosclerosis [18,19,20].

A major question in the understanding of laminopathies is how the molecular defect in the lamin A gene translates into disease symptoms. Laminopathic mutations interfere with the functions of the nuclear lamina leading to delayed or aberrant mitosis [21] and defects in epigenetic control [22]. The molecular defects in progerin leads to permanent farnesylation of the protein and inhibitors of farnesyl transferase can rescue some of the defects in cells expressing progerin [21].

Here, we investigated the role of serine 22 phosphorylation in both lamin A and progerin functions. We found that progerin is defective for serine 22 phosphorylation in interphase but the defect can be corrected by farnesyl transferase inhibitors or mutations that prevent farnesylation. Further serine 22 phosphorylation of progerin can be stimulated by a mutation that prevents head to tail interactions in lamin A. Intriguingly, progerin mutants that undergo serine 22 phosphorylation as well as a phosphomimetic S22D lamin A mutant form intranuclear lamin droplets. CDK inhibitors inhibit serine 22 phosphorylation of lamin A, increasing the levels of this protein accelerating the entry in senescence of fibroblasts from progeria patients. We present a new model of progeria where permanent farnesylation leads to defects in serine 22 phosphorylation. We propose that lack of phosphory-lation at serine 22 compromises phase separation of solid lamin A polymers, leading to accumulation of a fibrous lamin A structure that alters the functions of both the nuclear lamina and nucleoplasmic lamin A.

## RESULTS

### Intranuclear distribution of progerin depends on serine 22 phosphorylation

**Figure 1:**
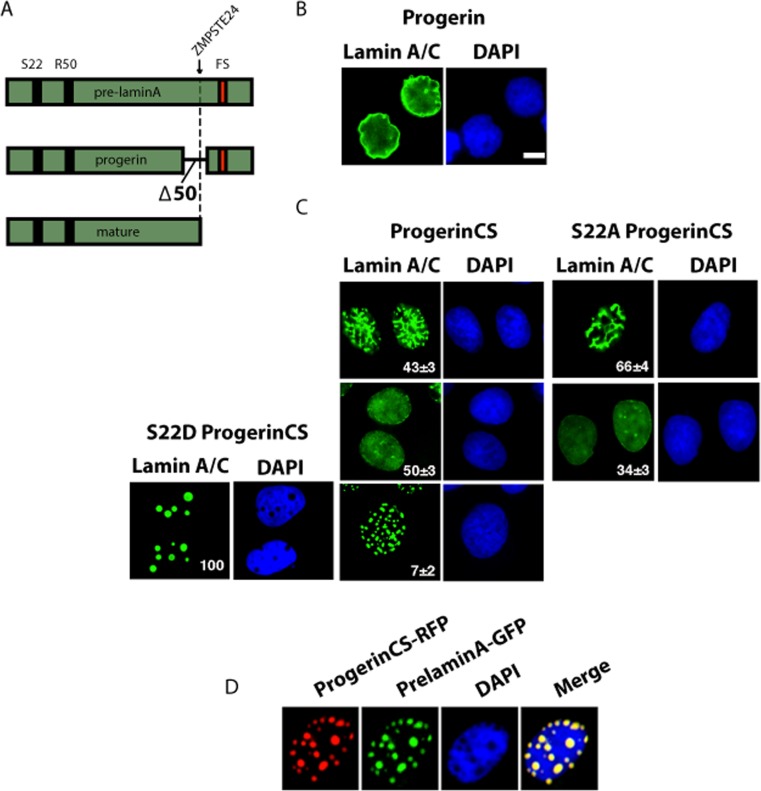
Distinct patterns of nuclear localization of progerin and non-farnesylated progerin (progerin CS) (**A**) Schematic representation of prelamin A alleles cloned into retroviral vectors and used in this study. FS= farnesylation site. Note the ZMPSTE24 cleavage site (arrow) that is absent in progerin. (**B-C**) Immunofluorescence for lamin A/C of U-2 OS cell expressing progerin or progerin CS with wild type S22 or mutations S22A or S22D. Cells were fixed 5 days after infection. The percent and standard deviation (S.D) of cells having each pattern is indicated at the bottom right of each panel. (**D**) Immunofluorescence images of U-2 OS cell coexpressing progerin CS fused with Red fluorescent protein (RFP) and prelamin A fused with GFP. Magnification = 10 μm.

To study the biology and turnover of progerin, the lamin A mutant protein responsible for Hutchinson-Gilford progeria syndrome, we used retroviral vectors that express the wild type prelamin A, mature lamin A (mimicking the protein obtained after cleavage by ZMPSTE24) and mutant derivatives (Figure 1A). Expression of progerin in U-2 OS cells leads to an accumulation of this protein in the nuclear envelope and deformation of the nucleus (Figure 1B). Mutating the cysteine residue in the farnesylation site of progerin to serine (Progerin CS) abolished its ability to induce nuclear deformation and led to three distinct patterns of intranuclear distribution of progerin (Figure 1C). Most cells have a homogenous staining in the nucleoplasm and the nuclear periphery (Figure 1C, center). This pattern is typical of endogenous lamin A staining reported in HeLa cells and reflects functions of lamin A both at the nuclear envelope and the nucleoplasm [5]. A large proportion of the cells display an intranuclear reticulum mainly composed of lamin rods and 7% of the cells have distinct foci (Figure 1C, top and bottom respectively). Mutation of serine 22 to alanine (S22A) blocked phosphorylation at this residue and changed the intranuclear distribution of the protein. Most cells showed either the rods pattern or homogenous staining but foci were entirely absent (Figure 1C). Mutation of serine 22 to aspartic acid (S22D) to mimic phosphorylation had a dramatic consequence for Progerin CS distribution and 100% of the cells displayed round foci (Figure 1C). These patterns suggest that Progerin CS foci are associated to serine 22 phosphorylation.

We were not able to detect endogenous lamin A in the nuclear lamina in cells with foci of Progerin CS probably because non-farnesylated progerin transits through the nuclear lamina, interacts with lamin A and carries it to the foci. To confirm the idea that Progerin CS can interact with wild type lamin A and recruit it to foci, we cotransfected U-2 OS cells with GFP-pre-lamin A and RFP-progerin CS and verified their localization using fluorescence microscopy. We found that non-farnesylated progerin and lamin A-GFP colocalized in foci (Figure 1D).

**Figure 2:**
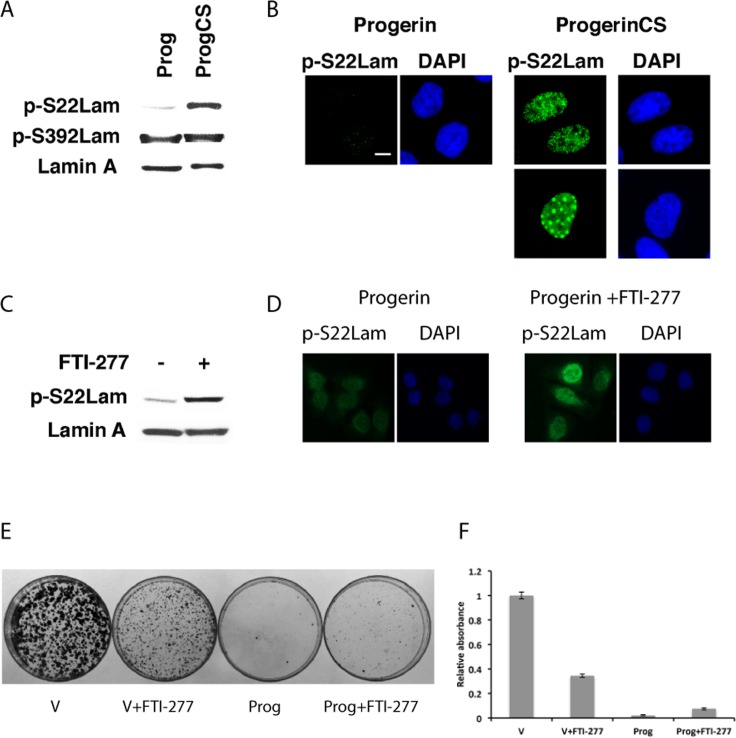
Intranuclear progerin foci contain serine 22 phosphorylated progerin (**A**) Immunoblots for phosphorylated lamin A at serines 22 and 392 in U-2 OS cells expressing progerin (Prog) or progerin CS (ProgCS). (**B**) Immunofluorescence for phosphoserine 22 lamin A (p-S22Lam) in U-2 OS cells expressing progerin or progerin CS. Magnification = 10 μm. (**C**) Immunoblot for phosphoserine 22 lamin A in U-2 OS cells expressing progerin and treated with 3 μM FTI-277 or vehicle. (**D**) Immunofluorescence for phosphoserine 22 lamin A in cells expressing progerin and treated with 3 μM FTI-277 or vehicle. (**E**) Colony assays of U2-OS cells expressing the indicated vectors and treated with 3 μM FTI-277 or vehicle. (**F**) Quantification of the colony assay in (**E**).

We next studied the phosphorylation of progerin and progerin CS at serines 22 and 392 in U-2 OS cells using phospho-specific antibodies. In non-synchronized cells, little progerin is phosphorylated at serine 22 but phosphorylation at serine 392 was readily detectable (Figure 2A and B). Progerin CS was phosphorylated at serine 22, displaying two staining patterns of localization (homogenous staining in most of the cells and foci) (Figure 2B). Although progerin was poorly phosphorylated at serine 22, treatment with the farnesyl transferase inhibitor FTI-277 increased its S22 phosphorylation (Figure 2C and D). Taken together the results show that farnesylation prevents efficient phos-phorylation of lamin A at serine 22 in interphase cells and suggest that one role for cleavage and defarnesylation of lamin A is to facilitate its phosphorylation at serine 22.

Since FTI-277 can correct the farnesylation defect of progerin and improve its phosphorylation at serine 22, we next investigated its effects on cell growth. Consistent with previous report [23], FTI-277 at 3 μM did not significantly improve the proliferation defect imposed by overexpression of progerin in U-2 OS cells (Figure 2E and F). In fact, FTI-277 reduced the proliferation of control U-2 OS cells in this assay.

### Depolymerization cooperates with defarnesylation to stimulate serine 22 phosphorylation of lamin A

Inhibition of lamin A farnesylation does not completely abolish its incorporation into the nuclear lamina [24,25]. In addition to farnesylation, lamin A uses head to tail interactions to form polymers and localize to the nuclear lamina [13]. It is also known that farnesylated proteins require a second signal for stable association to membranes [26]. Consistent with this, progerin in the nuclear lamina is farnesylated but also polymerized, suggesting that to control progerin levels and localization, in addition to defarnesylation the protein must be depolymerized. To verify this hypothesis, we created lamin A and progerin mutants in which the arginine at position 50 was replaced by a proline. This arginine in the conserved rod domain end of lamin A was shown to be critical for polymer formation [27]. Consistent with a previous report [27], R50P lamin A was not as well expressed as wild type lamin A and in some cells it did not form a continuous polymeric lamina as the wild type protein, but accumulated as foci in the nuclear envelope (Figure S1A). Introducing the phosphomimetic S22D mutation into lamin A led to the formation of nuclear foci in most cells both in the nucleoplasm and the nuclear envelope (Figure S1A).

Staining cells expressing these constructs with the anti-phosphoserine 22 antibody revealed a nucleoplasmic signal. Of note, none of the lamin A at the nuclear envelope was positive for serine 22 phosphorylation except the few foci at the nuclear envelope formed by R50P lamin A (Figure S1A). On immunoblots, a fraction of R50P prelamin A migrated at a higher molecular weight than mature lamin A (Figure S1B) suggesting that this mutation partially impairs maturation by ZMPSTE24. The phosphomimetic S22D lamin A migra-tes as mature lamin A (Figure S1B). Taken together, the results are consistent with the idea that defarnesylation promotes S22 phosphorylation in interphase cells.

**Figure 3:**
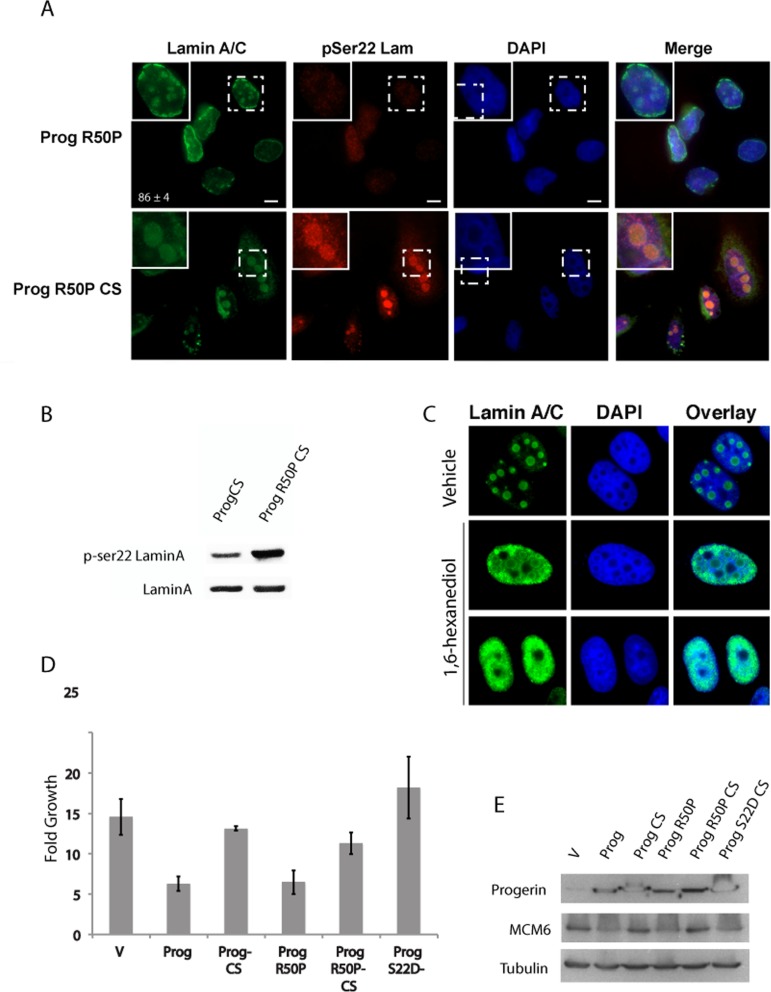
The R50P mutation increases serine 22 phosphorylation of non-farnesylated progerin (**A**) Immunofluorescence with anti-lamin A/C and anti-serine 22 phosphorylated lamin A in U-2 OS cells expressing the indicated proteins. (**B**) Phosphoserine 22 lamin A immunoblots in U-2 OS cells expressing the indicated proteins. (**C**) Immunofluorescence with anti-lamin A antibody in U-2 OS cells expressing Progerin R50P CS and treated with 1% hexanediol or vehicle. (**D**) Growth analysis of U-2 OS cells expressing the indicated proteins. (**E**) Anti-MCM6 and anti-progerin immunoblots in U-2 OS cells expressing the indicated proteins.

Next we performed a similar mutational analysis in progerin. Progerin R50P localized mainly in the nuclear envelope similar to lamin A R50P. In contrast, Progerin CS with the R50P mutation formed intranuclear foci (Figure 3A) similar to those formed by S22D progerin CS (see Figure 1C) and reminiscent of liquid droplets formed by proteins with low complexity domains [28].

Of note, lamin A contains several regions of low complexity according to DisEMBL [29], a method based on neural networks that predicts these regions to several definitions of protein disorder (Figure S2). Foci at the nuclear envelope formed by R50P progerin were not associated with phosphory-lation at serine 22 (Figure 3A). However, intranuclear R50P Progerin CS foci were stained by the phosphospecific antibody against S22 phospholamin A (Figure 3A) and immunoblots show that the R50P mutation increased the fraction of progerin CS phosphorylated at serine 22 (Figure 3B). Note that Progerin CS formed intranuclear rods or foci only in around 50% of the cells (Figure 1C) indicating that the R50P mutation dramatically alters the behavior of this protein. Foci formed by R50P progerin CS are not stained by DAPI and look like nucleoli. However, R50P progerin CS does not colocolize with the nucleolar marker fibrillarin (Figure S3). Interestingly, R50P progerin CS foci were disrupted by treatment with 1% 1,6-hexanediol, an aliphatic alcohol that disrupts weak hydrophobic interactions (Figure 3C) [30]. These results are consistent with a model where progerin is resistant to serine 22 phosphorylation during interphase. Cleavage and defarnesylation facilitates serine 22 phospho-rylation, while the R50P mutation alone does not. However, combining defarnesylation with the R50P mutation dramatically enhances serine 22 phosphorylation of progerin probably because the R50P mutation disrupts the head to tail interactions that retain progerin CS and mature lamin A in the nuclear envelope in a way inaccessible to serine 22 kinases. Another interesting feature is that R50P progerin CS does not form rods in the nucleoplasm as progerin CS (Figure 1C and Figure 3A) suggesting that progerin CS can still form polymeric aggregates in the nucleoplasm and that the R50P mutation prevents this aggregation.

Next we evaluated the effects of R50P and CS mutations on the ability of progerin to inhibit cell growth in the tumor cell line U-2 OS. The CS mutation, but not the R50P mutation, prevented growth inhibition by progerin (Figure 3D). This is consistent with the data showing that the R50P mutation alone cannot rescue the S22 phosphorylation and localization defects of progerin. The growth phenotype correlated with the expression of the E2F target gene and DNA replication protein MCM6 (Figure 3E).

### Interphase lamin A S22 kinases

**Figure 4:**
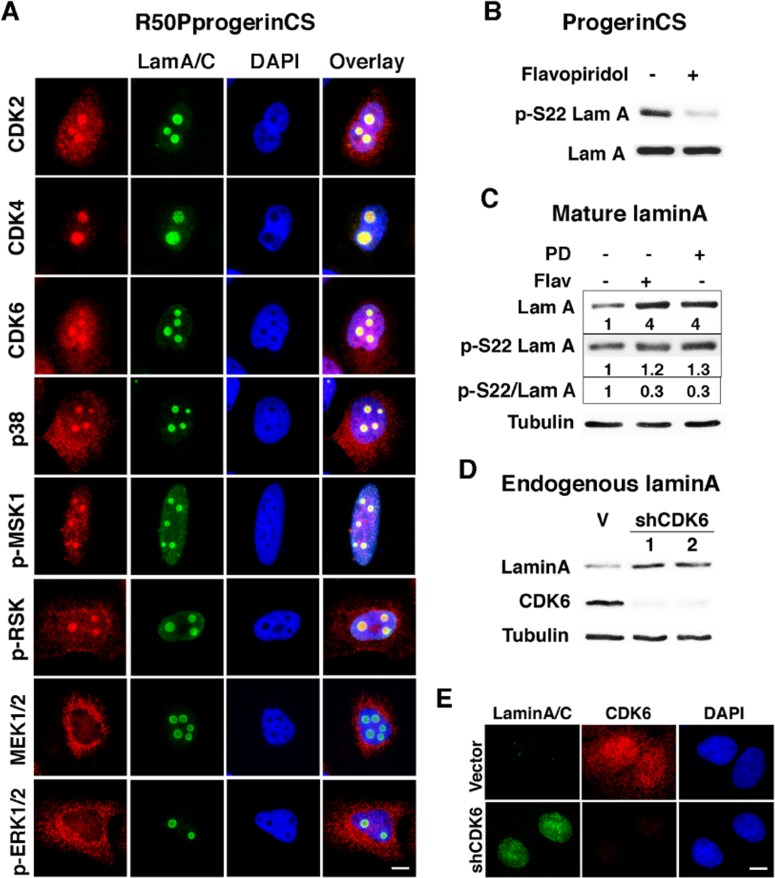
Flavopiridol sensitive kinases control serine 22 phosphorylation of non-farnesylated progerin and mature lamin A (**A**) Immunofluorescence with anti-lamin A/C (LamA/C, green) and antibodies against indicated kinases (red) in U-2 OS cells expressing R50P mutant of non-farnesylated progerin (R50PprogerinCS). (**B**) Phosphoserine 22 lamin A immunoblots in U-2 OS cells expressing non-farnesylated progerin (ProgerinCS) treated with 100 nM flavopiridol or vehicle for 2 days. (**C**) Lamin A and phosphoserine 22 lamin A immunoblots and phosphoserine 22 lamin A/lamin A (p-S22 LamA) quantified immunoblots and expression ratio estimation of phosphoserine 22 lamin A/lamin A (pS22/LamA) in U-2 OS cells expressing mature lamin A treated with 1 μM PD0332991 (PD) or 100 nM flavopiridol (Flav) or vehicle for 2 days. (**D**) Lamin A and CDK6 immunoblots in U-2 OS cells expressing shCDK6s or a control shRNA (V). (**E**) Immunofluorescence for lamin A/C and CDK6 in cells as in (D). Scale bar: 10 μm.

The phosphorylation of serine 22 of lamin A is a function of CDK1 during mitosis [14]. However, in HeLa cells, anti-phosphoserine 22 lamin A antibody labels interphase cells without CDK1 activity [16]. To investigate which kinases may affect intranuclear lamin A during interphase, we took advantage of the foci formed by the R50P progerin CS mutant that are easily visualized with anti-lamin A/C antibodies. Serine 22 is part of a SPTR motif recognized by proline directed kinases of the CDK/MAP kinase families. In agreement, we found multiple CDK/MAP kinases colocalized with R50P progerin CS in nuclear foci. They include CDK2, CDK4, CDK6 and p38 MAPK but not MEK or ERK (Figure 4A). Unexpectedly, MSK1 and RSK also localized to these foci, suggesting that R50P progerin could be phosphorylated at other sites by these kinases as well. In cells expressing R50P progerin CS, the pan CDK inhibitor flavopiridol [31] suppressed serine 22 phosphorylation (Figure 4B). Flavopiridol did not change the total levels of progerin CS as measured with an anti-lamin A antibody. The fraction of progerin CS that is phosphorylated should correspond to the fraction that forms foci in the nucleoplasm (around 7% of the cells in Figure 1C). Hence, if inhibition of phosphorylation stabilized progerin, it will be difficult to observe this effect in progerin CS expressing cells where still a large fraction of the protein is associated to the nuclear lamina or form intranuclear aggregates.

To investigate whether inhibition of phosphorylation affects lamin A levels, we expressed mature lamin A (normally lamin A is first expressed as prelamin A which is then processed by ZMPSTE24 into the mature protein). Using this construct we can now visualize that both flavopiridol and the CDK4/6 inhibitor PD0332991 increased mature lamin A levels (Figure 4C) suggesting that CDK-dependent phosphorylation of lamin A controls its levels. As expected, both drugs inhibited lamin A phosphorylation at serine 22 as measured by normalization phospho-serine 22 lamin A levels on total lamin A levels (Figure 4C). To further investigate the role of endogenous CDK activity on endogenous lamin A levels in interphase, we used two validated shRNA against CDK6. Reducing CDK6 levels increased lamin A levels as assessed by immunoblots (Figure 4D) or immunofluorescence (Figure 4E). These results suggest that mature lamin A levels are controlled by CDK-dependent serine 22 phosphorylation in interphase.

### Farnesyl transferase inhibitors increase serine 22 phosphorylation of endogenous progerin

**Figure 5:**
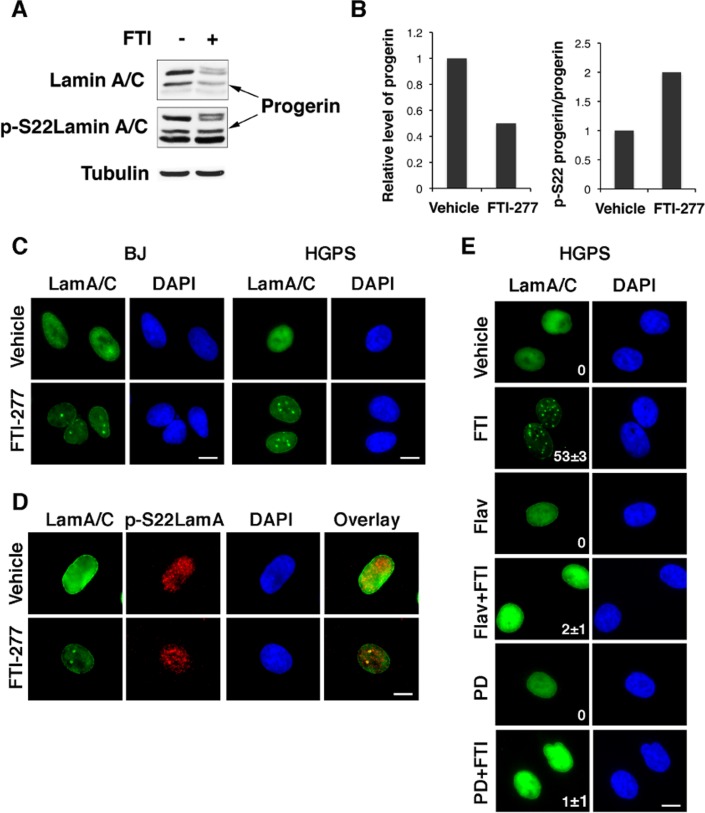
Inhibition of endogenous progerin farnesylation controls its phosphorylation and levels in fibroblasts from progeria patients (**A**) Immunoblots for lamin A/C and phosphoserine 22 lamin A in fibroblasts from HGPS patients. (**B**) Normalizing phosphoserine 22 lamin A levels to progerin levels using the data in (A). (**C**) Immunofluorescence for lamin A/C in BJ young fibroblasts and HGPS fibroblasts after treatment with 3 μM FTI-277 or vehicle. (**D**) Immunofluorescence for lamin A/C and phospho serine 22 lamin A in HGPS fibroblasts after treatment with 3 μM FTI-277 or vehicle. (**E**) Immunofluorescence for phosphoserine 22 lamin A in HGPS fibroblasts after treatment with 3 μM FTI-277 and/or 60 nM flavopiridol or 1μM PD0332991 (PD).

Next we studied whether preventing farnesylation could increase the phosphorylation of endogenous progerin in cells obtained from patients with HGPS. Treatment with FTI-277 decreased the levels of endogenous progerin so we normalized the signal of serine 22 phosphorylation on the levels of progerin in cells. After this normalization, it is clear that FTI-277 increased serine 22 phosphorylation of endogenous progerin (Figures 5A and B). We conclude that absence of farnesylation facilitates the phosphorylation of endogenous lamin A at serine 22 during interphase.

To determine whether inhibition of farnesylation affected the localization of endogenous progerin, we used immunofluorescence in normal and HGPS fibroblasts treated with FTI-277. Both wild type lamin A and progerin localized to intranuclear foci after treatment with FTI-277 (Figure 5C) and these foci were stained by the anti-phospho serine 22 lamin A antibody (Figure 5D). This phosphorylation was important to control the overall levels of progerin, since treatment of HGPS cells with FTI-277 decreased progerin levels and induced intranuclear foci but both flavopiridol and PD0332991 inhibited the formation of foci and increased the levels of progerin (Figure 5E).

**Figure 6:**
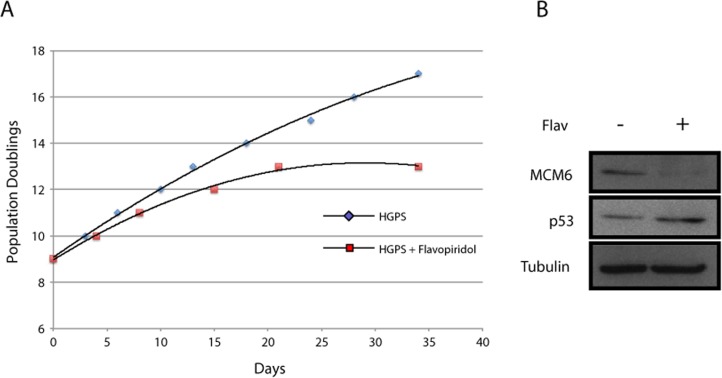
CDK inhibitor accelerates senescence in HGPS fibroblasts (**A**) Population doublings calculated after serial passage of the cells starting at population doubling 9 and treated with vehicle or 60 nM flavopiridol for the indicated amount of days. (**B**) Immunoblots in cells as in (**A**) for the indicated proteins.

It is known that older cells express higher levels of CDK inhibitors such as p16INK4a [32] and p21 [33]. It is thus possible that abnormalities in HGPS increase with age or accumulate in tissues with high turnover because concomitant expression of CDK inhibitors (CKI) in these situations further impairs serine 22 phosphorylation of progerin. In agreement with this model, we recently reported that mutating serine 22 to alanine in progerin increases the ability of the protein to induce cellular senescence [34]. We thus treated HGPS cells with the CDK inhibitor flavopiridol and evaluated the number of population doublings required to attain senescence. We found that the CDK inhibitor accelerated the senescence process in HGPS fibroblasts (Figure 6A). Consistent with the senescence phenotype, cells treated with flavopiridol have lower expression of the E2F target gene MCM6 and high expression of p53. This result suggests a pathological synergism between expression of progerin and senescence-associated expression of CDK inhibitors.

## DISCUSSION

**Figure 7:**
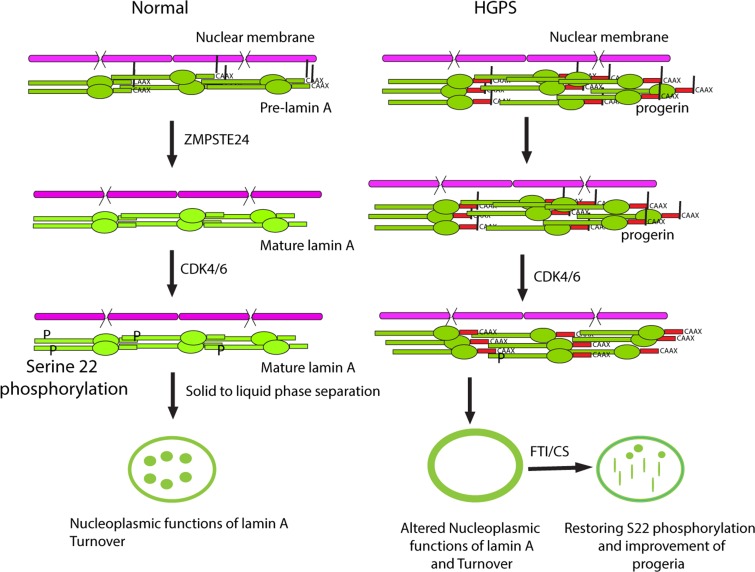
Cleavage and defarnesylation of lamin A allows serine 22 phosphorylation, a modification that promotes a solid to liquid phase separation of lamin polymers This phase separation can make soluble lamin A available for nucleoplasmic functions and allows lamin A turnover, regulating the overall size and stiffness of the envelope. Progerin is not defarnesylated and is resistant to phosphorylation at serine 22, compromising its solubilization, nucleoplasmic functions and turnover. FTI-277 or the mutation of the farnesylation site (CS) partially correct the defects in progerin but the solid to liquid transition is defective and intranuclear progerin forms mostly fibrous rods. New treatments are required for a full normalization of lamin A in progeria.

We propose a dynamic model of lamin A functions, localization and turnover controlled by defarnesylation and serine 22 phosphorylation (Figure 7). The first step for lamin A turnover is its defarnesylation that normally occurs by cleaving the protein upstream the far-nesylation site by the protease ZMPSTE24. This cleavage facilitates serine 22 phosphorylation and the translocation of lamin A to the nucleoplasm. Non-defarnesylated lamin A mutants such as progerin become resistant to serine 22 phosphorylation and as a consequence they are defective for lamin A translocation from the nuclear lamina to the nucleo-plasm. Since nucleoplasmic lamin A plays a role in several nuclear processes, these functions may be defective in progeria patients. We thus propose a specific biochemical role for lamin A defarnesylation that can be partially corrected using FTIs.

However, inhibition of farnesylation cannot correct all defects associated to progerin expression. First, treatment with FTI does not prevent the ability of progerin to inhibit cell growth (this study and [23]). Second, FTI does not inhibit senescence and DNA damage in cells from HGPS [35]. Third, in a mouse model of HGPS, FTI improved many disease symptoms but did not totally reverse them [36]. Fourth, mutations of the farnesylation site in progerin promotes the detachment of a fraction of progerin from the nuclear envelope but most of the protein accumulates as rods resembling fibrillar aggregates in the nucleoplasm (Figure 1). Finally, the extra-uncleaved sequence present in progerin but not in mature lamin A can mediate protein-protein interactions that prevents its solubilization even after defarnesylation [37].

Mature lamin A remains in the nuclear lamina after defarnesylation by head to tail contacts between lamin filaments [13] and by interactions with proteins of the inner nuclear membrane [38]. Head to tail lamin A interactions can be disrupted by the mutation R50P found in patients with a laminopathy [27]. We found that introduction of the R50P mutation dramatically changes the behavior of progerin when farnesylation is inhibited. The R50P mutation promotes the formation of spherical aggregates of progerin CS. These aggregates are reminiscent of liquid droplets formed by several proteins that contain low complexity domains and can engage in multivalent interactions [28]. The R50P mutation also prevents the formation of fibrous nuclear aggregates of progerin CS and increases the phosphorylation of defarnesylated progerin at serine 22. Taking together, our data suggest that full normalization of the biochemical properties of progerin will require not only farnesylation inhibition but also a treatment that inhibits its polymeric interactions at the nuclear envelope.

CDK1 phosphorylates lamin A at serine 22 in the nuclear envelope as part of the mitotic nuclear envelope disassembly pathway [14]. CDK4/6 could in principle also act in the nuclear envelope and their colocalization with intranuclear lamin A foci simply reflects a dynamic phase separation of phosphorylated lamin A that attracts many interacting proteins with it. An important discovery is that progerin either in the nuclear envelope or in nucleoplasmic rods is not phosphorylated at serine 22, suggesting that CDK4/6 kinases cannot phosphorylate polymeric aggregates of progerin. Farnesylation inhibition and the R50P mutation that prevents head to tail interactions of progerin both promote serine 22 phosphorylation. This phosphory-lation controls lamin A stability since inhibition of phosphorylation using CDK4/6 inhibitors increase lamin A levels. Although CDK4/6 mediated interphase phosphorylation of progerin is defective, progerin can be phosphorylated at serine 22 during mitosis [34] probably because other mitosis associated events help to expose serine 22 in lamin A. According to our model, serine 22 phosphorylation controls lamin A biophysical properties and turnover during interphase, explaining why progerin accumulates in the nuclear lamina at very high levels.

In cells taken from HGPS patients, progerin accumulates in older cell cultures in association with nuclear abnormalities and mitotic defects [21,39]. A new paradigm to understand the relationship between age and disease involves a liquid to solid phase transition by proteins that contain low complexity domains [40]. In addition, phosphorylation stimulates phase transition in proteins that form liquid droplets [28]. We show here that lamin A and progerin contain low complexity domains and they can form liquid droplets or rod-like aggregates when specific mutations are engineered in the proteins. Based on fluorescent studies of lamin A coupled to GFP in nuclei undergoing deformation, it has been suggested that lamin A forms a continuous two-dimensional solid [11]. Consistent with this model, studies of lamin A dynamics using fluorescence recovery after photobleaching indicates that lamin A form very stable structures [8]. We propose that serine 22 phosphorylation promotes phase separation of lamin A polymers and the proper partition of the protein between the nuclear lamina and the nucleoplasm. Consistent with this idea, a phosphomimetic mutation at serine 22 in progerin induces the formation of spherical aggregates of unfarnesylated progerin. We observed similar albeit smaller intranuclear lamin A aggregates in HGPS cells after treatment with FTIs, which stimulate serine 22 phosphorylation of progerin. The functional role of these lamin A droplets remain to be characterized but they may play a role to facilitate the intranuclear distribution and functions of lamin A.

In conclusion, we show that defects in defarnesylation of prelamin A impair serine 22 phosphorylation. Hence, mutations in ZMPSTE24 leading to accumulation of farnesylated prelamin A and the progerin mutation have a common molecular defect that could explain why they are both associated to cellular senescence and aging [41,42]. The molecular mechanism linking de-farnesylation to serine 22 phosphorylation also suggests a self-reinforcing aging mechanism. In this model, cellular aging could start by small amounts of progerin accumulation due to errors in splicing, a phenomenon observed in several old human tissues [43,44,45,46]. CKIs induced by short telomeres or other senescence-inducing stresses prevent the turnover of progerin by inhibiting CDK-dependent S22 phosphorylation. In turn, accumulation of progerin can induce CKIs expression by interfering with telomere functions, the regulation of gene expression or DNA repair. According to this view, HGPS is a disease that magnifies and accelerates the process of aging and treatments to improve this condition could also improve health in the old population.

## MATERIALS AND METHODS

### Cells and compounds

Human osteosarcoma U-2 OS cells (HTB-96, ATCC), BJ fibroblasts (a gift from S. W Lowe) and fibroblasts from progeria patient (HGADFN003, Progeria Foundation) were cultured in Dulbecco's modified Eagle's medium (Wisent, St-Bruno, QC) supplemented with 10% (for U-2 OS and BJ) or 15% (for HGADFN003) fetal bovine serum (FBS) (Wisent). Flavopiridol was from (Selleckchem, http://www.selleckchem.com/). PD0332991 and FTI-277 were purchased from Sigma-Aldrich.

### Retroviral vectors and retroviral-mediated gene transfer

Retroviral vector pLPC is from S. W. Lowe. pLPC wtlaminA, pLPC progerin, pLPC progerinCS, pLPC S22AprogerinCS were described in [34]. S22D point mutation was introduced into progerinCS and wild type prelamin A by site-directed mutagenesis using forward primer 5′-AGCTCCACTCCGCTGGACCCCACCCG CATCACC-3′ and reverse primer 5′-GGTGATGCG GGTGGGGTCCAGCGGAGTGGAGCT-3′. R50P point mutation (CGC→CCC) was introduced into progerin, progerinCS and wild type prelamin A expression vectors by subcloning synthetic fragments. Mature lamin A was constructed using wild type lamin A as a template and the primers: forward 5′-CTTGGATCCACCATGGAGACCCCGTCCCAGCGGCG-3′ and reverse 5′-CTTCTCGAGTTAGAGGTAGG AGCGGGTGACCAGATTGTC-3′. MLPpuroshCDK6s (Acevedo et al. Cancer Res. in press). Retroviral-mediated gene transfers were done as in [47].

### Immunoblotting and immunoprecipitations

To prepare total protein lysates, cells were collected by trypsinization, washed with phosphate-buffered saline (PBS), lysed in sodium dodecyl sulfate (SDS) sample buffer (60 mM Tris-HCl, pH 6.8, 10% glycerol, 2% SDS, and 5% β-mercaptoethanol), and boiled for 5 min. For Western blotting, 25 μg of total protein were separated on SDS-polyacrylamide gel electrophoresis gels and transferred to Immobilon-P membranes (Millipore, Bedford, MA). We used the following primary antibodies: anti-LaminA/C (2032, 1:1000, Cell Signaling), anti-phospho-Ser22LaminA (2026, 1:1000, Cell Signaling), anti-phospho-Ser392LaminA (A0503, 1:500, Asaybiotech), anti-progerin (13A4 ab66587, Abcan, 1:1000), anti-p38 (9212, 1:1000, Cell Signaling), anti-CDK6 (C21, SC-177, 1:250, Santa Cruz Biotechnology), anti-CDK2 (M-2, SC-163, 1:250, Santa Cruz Biotechnology), anti-MCM6 (provided by Dr. Heidebrecht, 1:10), anti-Tubulin (T6074, 1:5,000, Sigma). Signals were revealed after incubation with anti-mouse or anti-rabbit secondary antibodies coupled to peroxidase (Biorad) by using enhanced chemiluminescence (Amersham Pharmacia).

### Fluorescence microscopy

Cells were plated on coverslips at least 24h prior to fixation with 4% paraformaldehyde in PBS for 15 minutes at room temperature. After washing with PBS, cells were permeabilized using 0.2% Triton X-100 in PBS–3% bovine serum albumin (BSA) solution for 5 min. The cells were then washed three times with PBS–3% BSA and incubated for 1-2 h at room temperature with the primary antibody: anti-laminA/C (636) (sc-7292, 1:500, Santa Cruz Biotechnology), anti-phospho-Ser22LaminA (2026, 1:200, Cell Signaling), anti-CDK4 (DC5156, 2906, 1:100, Cell Signaling), anti-CDK2 (M-2, SC-163, 1:200, Santa Cruz Biotechnology), anti-CDK6 (C-21, SC-177, 1:200, Santa Cruz Biotechnology), anti-p38 (9212, 1:200, Cell Signaling), anti-phospho-MSK1 (9595, 1:200, Cell Signaling), anti-phospho-RSK (9341, 1:200, Cell Signaling), anti-MEK1/2 (9122, 1:200, Cell Signaling), anti-phospho-ERK1/2 (4370, 1:400, Cell Signaling) diluted in PBS/BSA. Next, cells were washed three times in PBS/BSA and incubated with the appropriate secondary antibody (1:2000, AlexaFluor 488 goat anti-mouse, AlexaFluor 568 goat anti-mouse, or AlexaFluor 488 goat anti-rabbit, Molecular Probes-Thermofisher-Scientific) for 1h at room temperature. Finally, cells were rinsed three times with PBS alone and once with PBS containing 300 nM DAPI for 10 minutes. Images from independent fields were captured with a fluorescence microscope and processed with Metamorph.

For superresolution structured illumination microscopy, cells were fixed as described above and stained with anti-lamin A/C antibody (as above) or anti-fibrillarin (C13C3, 2639, Cell Signaling Technology, 1:200). After washing they were stained with Alexa Fluor 568 goat anti-rabbit IgG (H+L) (A11029, Life Technologies, 1:2000) and Alexa Fluor 568 goat anti-rabbit IgG (H+L) (A11036, Life Technologies, 1:2000). Images were obtained with Super Resolution microscope Axio Observer Z1 ZEISS Elyra PS.1, SR-SIM, 3D PALM, STORM from Zeiss. We picked up images sets of 5 subsets each captured after rotating the grid by 5 degrees. A high resolution image was extracted from the raw data using the Structured Illumination Microscopy and the Maximum Intensity Projection algorithms at a lateral resolution (XY) of 120 nm and an axial resolution (Z) of 300 nm.

## SUPPLEMENTARY FIGURES


