# Supplementary material for: Permanent farnesylation of lamin A mutants linked to progeria impairs its phosphorylation at serine 22 during interphase
Source: Aging (Albany NY). 2016 Feb 21;8(2):366–81. doi: 10.18632/aging.100903 (PMC4789588; doi:10.18632/aging.100903)
Supplement: Supplementary file 1 [file aging-08-366-s001.pdf]

SUPPLEMENTARY FIGURES

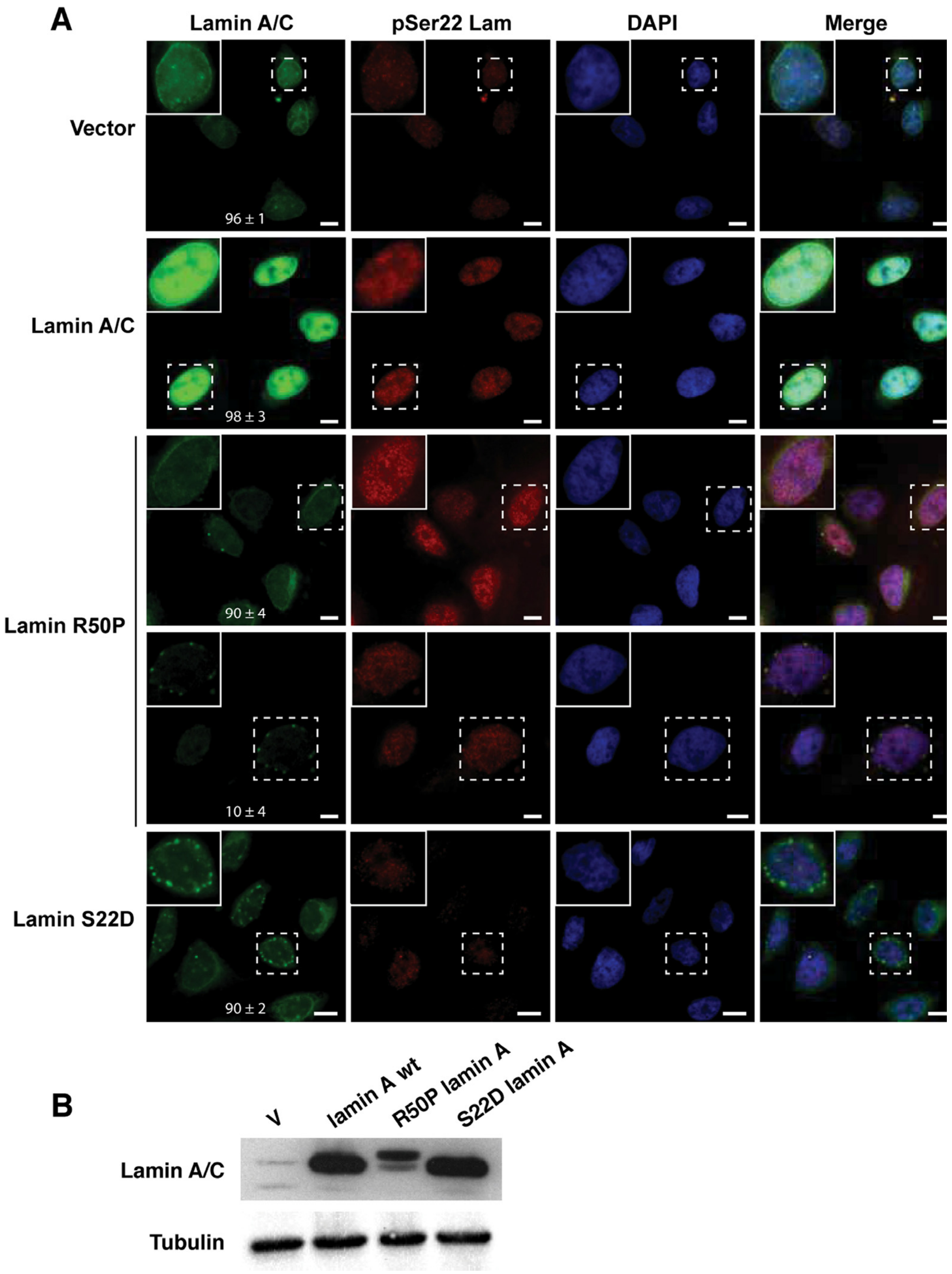

Figure S1. The polymerization defective R50P lamin A is resistant to defarnesylation. (**A**) Immunofluorescence with anti-lamin A/C and anti-serine 22 phosphorylated lamin A (pS22Lam) in U-2 OS cells expressing the indicated proteins. Magnification = 10 μm. (**B**) Lamin A/C immunoblots in U-2 OS cells expressing a control vector (V), lamin a wild type (wt) or either R50P or S22D lamin A mutants.

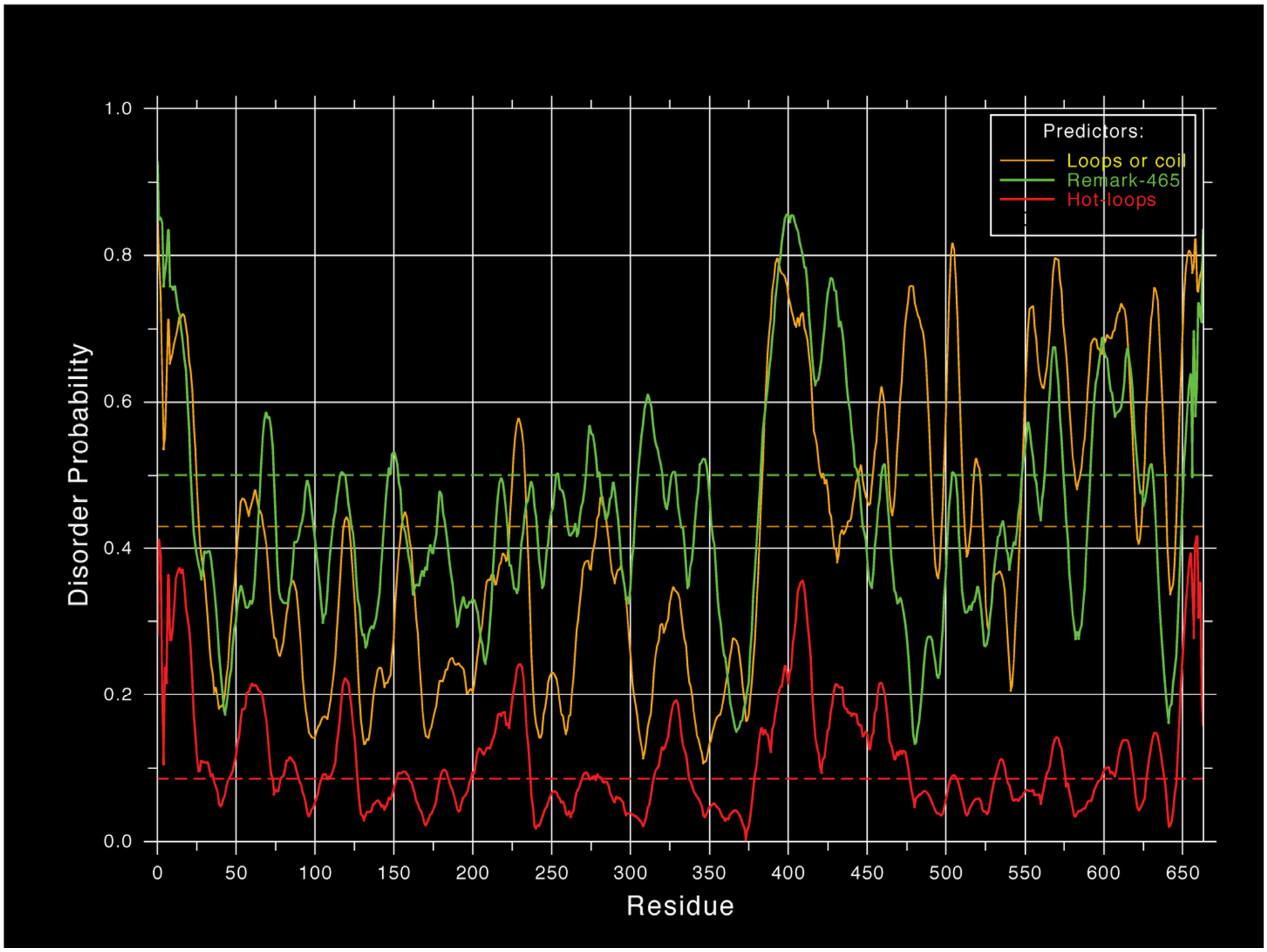

Figure S2. Low complexity domains in lamin A according to DisEMBL. Disordered regions are predicted according to three definitions. 1) Loop coils (orange) which is based on the fact that protein disorder is only found within loops. Loop assignment is necessary but not sufficient to predict disorder. 2) Hot loops (red) that refine loops coils by calculating the degree of mobility of the loops as predicted from the C-α temperature factors. 3) Missing coordinates in X-Ray data as defined by REMARK465 in the protein data bank (PDB, green). According to these three criteria lamin A contains several disordered regions in the N- and C-terminus.

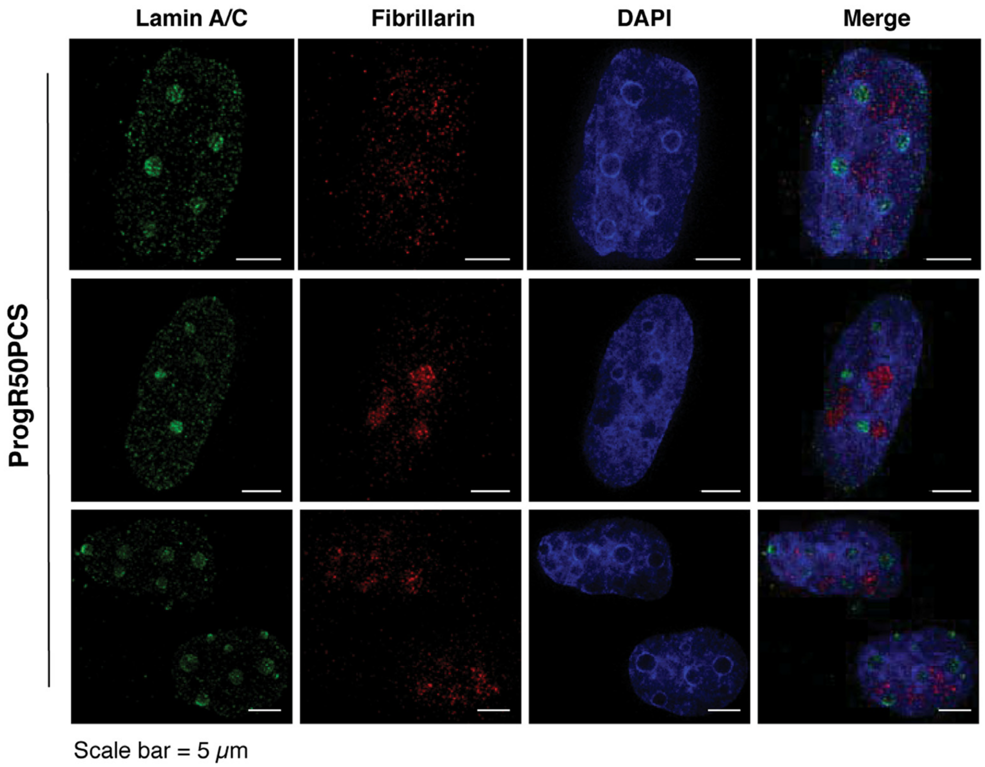

Figure S3. R50P progerin CS does not localize to nucleoli. Structure illumination microscopy of immunofluorescence staining using anti-lamin A/C and fibrillarin antibodies in U-2 OS cells expressing the indicated constructs. Scale bar: 5 μm.
